# One compound of saponins from *Disocorea zingiberensis* protected against experimental acute pancreatitis by preventing mitochondria-mediated necrosis

**DOI:** 10.1038/srep35965

**Published:** 2016-10-25

**Authors:** Rui Zhang, Li Wen, Yan Shen, Na Shi, Zhihua Xing, Qing Xia, Hai Niu, Wen Huang

**Affiliations:** 1 Laboratory of Ethnopharmacology, Regenerative Medicine Research Center, West China Hospital/West China Medical School, Sichuan University, Chengdu, China; 2 Department of Pediatric Gastroenterology, Children’s Hospital of Pittsburgh of UPMC and School of Medicine, University of Pittsburgh, Pittsburgh, PA, United States; 3 Department of Integrated Traditional Chinese and Western Medicine, West China Hospital, Sichuan University, Chengdu, Sichuan, China.; 4 College of Mathematics, Sichuan University, Chengdu, Sichuan, China

## Abstract

Acute pancreatitis (AP) is a painful inflammatory disorder of the exocrine pancreas, ranking as the most common gastrointestinal reasons for hospitalization with no specific therapy currently. Diosgenyl saponins extracted from natural products and diosgenin or its derivatives have been shown to exert anti-inflammatory effects in various diseases. However, the therapeutic effects of diosgenyl saponins from *Dioscorea zingiberensis* C. H. Wright in AP have not yet been determined. Five compounds were extracted and screened for taurocholate-induced necrosis in mouse pancreatic acinar cells. Particularly, 26-*O*-β-d-glucopyranosyl-3β, 22α, 26-trihydroxy-25(R)-furosta-5-en-3-*O*-[α-L-rhamnopyranosyl-(1 → 4)]-β-d-glucopyranoside (compound **1**) exhibited the best protective effects with no toxicity observed. Next, we showed compound **1** concentration-dependently inhibited necrotic cell death pathway activation and 2.5 mM compound **1** also prevented the loss of mitochondrial membrane potential, adenosine triphosphate production, and reactive oxygen species generation in mouse pancreatic acinar cells. Finally, we showed compound **1** protected against three clinically representative murine models of AP and significantly improved pancreatitis-associated acute lung injury. These data provide *in vitro* and *in vivo* evidence that one compound of diosgenyl saponins can be potential treatment for AP. This study suggests natural saponins may serve as fruitful sources for exploring/identifying potential therapies for inflammatory diseases.

Acute pancreatitis (AP) is a common inflammatory disease of the exocrine pancreas, ranking among the most common gastrointestinal reasons for hospitalization^1^. AP has an incidence of 30–50 per 100, 000 people per year worldwide^2^, mainly caused by gallstones or alcohol misuse^3^. Although majority of patients present as a mild self-limiting disorder, one out of five AP patients can develop a severe form with substantial morbidity, mortality and financial burden^1^,^3^. Over last two decades, cumulative mechanistic studies of AP suggest that the initial pancreatic injury starts within pancreatic acinar/ductal cells^4^,^5^,^6^ and mitochondria plays a central role in mediating disease severity and progression^7^,^8^. Pancreatitis-associated toxins, such as bile acids, cause mitochondrial inner membrane depolarization, adenosine triphosphate (ATP) depletion and excessive reactive oxygen species (ROS) production, and eventually necrotic cell death pathway activation^5^,^8^. The extent of necrosis is directly associated with disease severity in animal models^9^, whereas pancreatic necrosis is one of the key determinants of mortality in patients with AP^10^. Thus, the strategies for preventing pancreatic necrosis could be potentially beneficial treatment of AP, which currently has no specific drug therapy.

Steroidal saponins are well-known natural saponins from *Dioscorea zingiberensis* C. H. Wright, *Panax notoginseng*, *Smilax china*, *Polygonatum zanlanscianese, Asparagus officinalis* and so on^11^. The pharmacological activities of steroidal saponins, including anti-tumor^12^, anti-inflammatory^13^, anti-hypercholesterolemic^14^, anti-oxidant^15^, anti-viral^16^, neuroprotective^17^, have been widely explored and studied. Diosgenin, the core chemical structure from the extracts is often the key material used to synthesize steroid hormone drugs. We previously showed diosgenin and a series of its derivatives have anti-inflammatory effects on xylene-induced ear edema in mice^18^. Neither Diosgenyl saponins from *Dioscorea zingiberensis* C. H. Wright nor diosgenin/its derivatives have yet been studied in acute pancreatitis. In this study, we aim to determine the therapeutic effects of diosgenyl saponins from *Dioscorea zingiberensis* C. H. Wright using mouse pancreatic acinar cells and three clinically representative murine models of AP.

We firstly verified the chemical structures of five compounds isolated from *Dioscorea zingiberensis* C. H. Wright by ^1^H and ^13^C NMR spectra, compared with published literatures. We screened these five compounds on taurocholic acid sodium salt hydrate (NaT)-induced necrosis and we only found 26-*O*-β-d-glucopyranosyl-3β, 22α, 26-trihydroxy-25(R)-furosta-5-en-3-*O*-[α-l-rhamnopyranosyl-(1 → 4)]-β-d-glucopyranoside (compound **1**) exhibited more consistent and pronounced effects on protecting against NaT-induced necrosis at all tested concentrations with no toxicity observed. We further demonstrated that compound **1** concentration-dependently protected against necrotic cell death pathway activation with the maximal inhibitory effect at 2.5 mM and the same concentration (2.5 mM) prevented the loss of mitochondrial membrane potential (ΔΨm), ATP depletion and ROS production in mouse pancreatic acinar cells. Finally, we showed that compound **1** protected against three clinically representative models of AP. Remarkably, compound **1** also significantly improved pancreatitis-associated acute lung injury (ALI), which is one of the most common complications of AP and associated with higher mortality^10^,^19^. These data provide *in vitro* and *in vivo* evidence that diosgenyl saponins from *Dioscorea zingiberensis* C. H. Wright, particularly compound **1** can be potential treatment for AP.

## Results

### Compounds isolated from *Dioscorea zingiberensis* protected against NaT-induced necrosis

**Figure 1:**
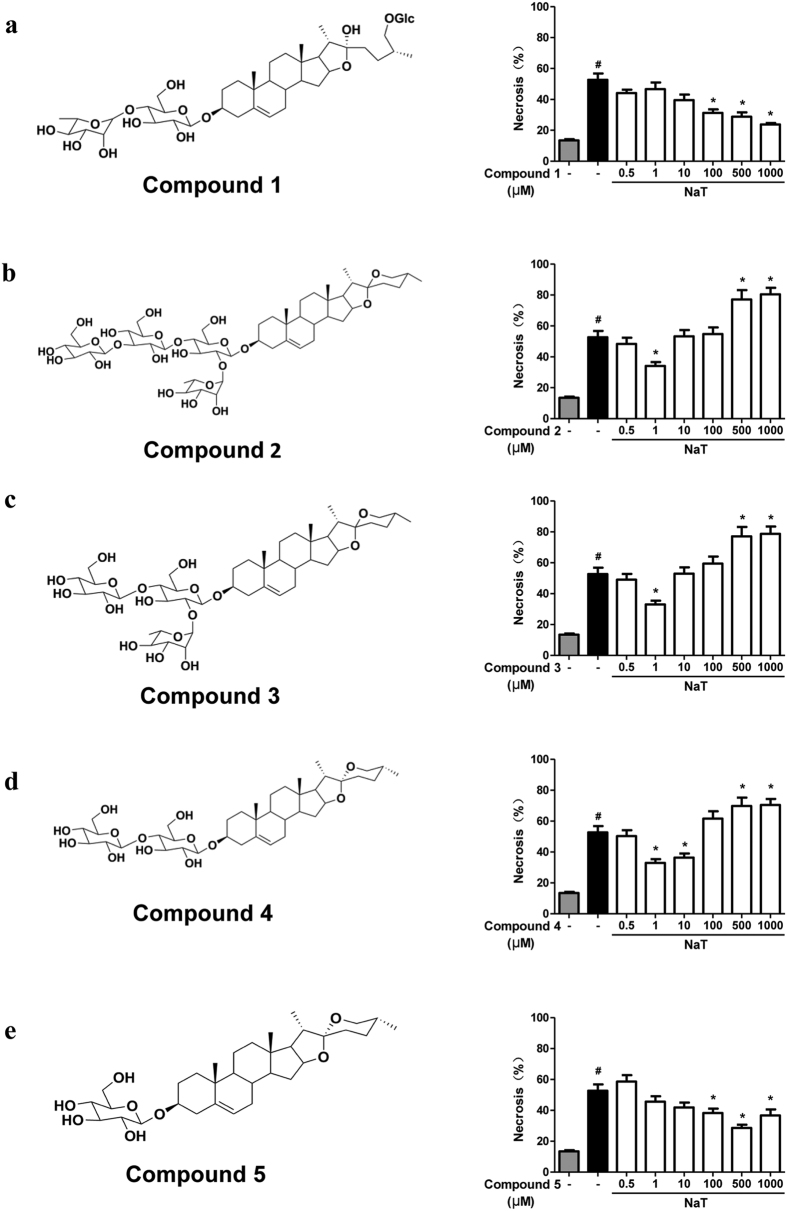
Structures of compound 1-5 and cytoprotective effects assessed by necrosis in mouse pancreatic acinar cells. **(a)** 26-*O*-β-d-glucopyranosyl-3β, 22α, 26-trihydroxy-25(R)-furosta-5-en-3-*O*-[α-L-rhamnopyranosyl-(1 → 4)]-β-d-glucopyranoside diosgenin, **(b)** zingiberensis saponin, **(c)** deltonin, **(d)** diosgenin diglucoside, **(e)** trillin. Necrosis (%) was calculated by propidium iodide (PI) positive staining divided by Hoechst 33342 positive staining × 100. Data were represented as mean ± S.E.M. and n ≥ 3 per condition. ^#^ and * p < 0.05 vs control and taurocholic acid sodium salt hydrate (NaT), respectively.

**Figure 2:**
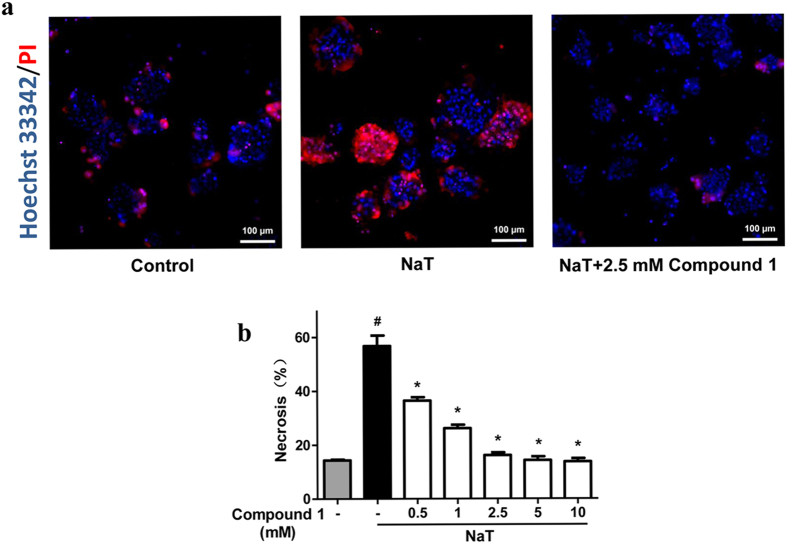
Compound 1 concentration-dependently protected against necrotic cell death pathway activation in mouse pancreatic acinar cells. (**a**) Representative images showing Hoechst 33342 (blue) and PI (red) staining from control, 5 mM NaT and 5 mM NaT treated with 2.5 mM compound **1**. (**b**) Necrosis (%) was calculated by PI positive staining divided by Hoechst 33342 positive staining × 100. Data were represented as mean ± S.E.M. and n ≥ 3 per condition. ^#^ and * p < 0.05 vs control and NaT, respectively.

Firstly, we identified chemical structures of compound **1–5** isolated from *Dioscorea zingiberensis*, using^1^H and ^13^C NMR spectra (Fig. 1). Compound **1** was identified as 26-*O*-β-d-glucopyranosyl-3β,22α, 26-trihydroxy-25(R)-furosta-5-en-3-*O*-[α-l-rhamnopyranosyl-(1 → 4)]-β-d-glucopyranoside by comparison of NMR data with those in the literatures^20^. Compounds **2**–**5** were characterized as we described earlier^12^, their experimental data were in complete agreement to zingiberensis saponin, deltonin, diosgenin diglucoside, and trillin, respectively. Structures of compounds **2**–**5** were consistent with the published reports^12^. We then screened cytoprotective effects of compounds **1–5**, using a well-established necrosis assay to assess plasma membrane rupture staining with propidium iodide (PI) in mouse pancreatic acinar cells. A wide range of concentrations of compound **1–5** (from 0.5 to 1000 μM) were evaluated (Fig. 1a–e). Compound **1** and **5** showed a concentration-dependently inhibitory effects on necrotic cell death pathway activation induced by 5 mM NaT, with no toxic effects observed at the higher concentrations (500 and 1000 μM); Compound **1** had more pronounced inhibitory effects than compound **5** (Fig. 1a,e). Compound **2**, **3** and **4** all significantly reduced necrotic cell death pathway activation at 1 or 10 μM, but also caused a significant increase of necrosis at 500 and 1000 μM (Fig. 1b–d). Since compound **1** exhibited more pronounced and consistent cytoprotective effects with the highest concentration only showing 50% inhibitory effect on necrosis, we then more closely investigated the effects of compound **1** at 0.5, 1, 2.5, 5 and 10 mM on necrotic cell death pathway activation induced by 5 mM NaT. Compound **1** concentration-dependently inhibited necrosis; at 2.5 mM showed a maximal inhibitory effect (Fig. 2a,b).

### Compound 1 prevented the loss of ΔΨm, ATP depletion, and ROS production

**Figure 3:**
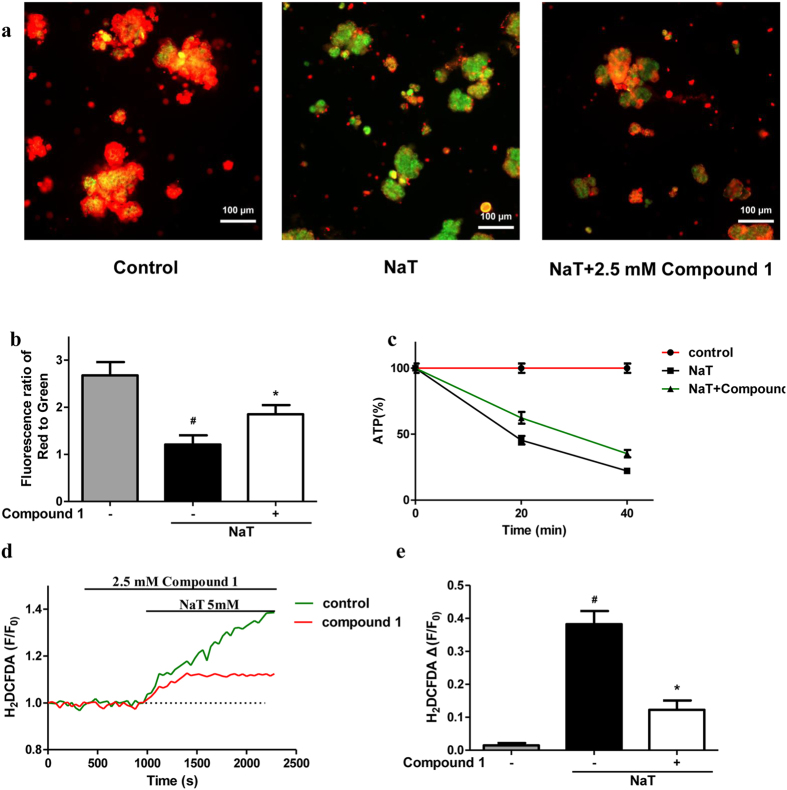
Compound 1 prevented the loss of ΔΨm, ATP depletion and ROS production induced by NaT in mouse pancreatic acinar cells. **(a)** Representative images of JC-1 staining from control, NaT and NaT plus 2.5 mM compound **1,** JC-1 manifests red fluorescence at high membrane potential while green fluorescence appears at low membrane potential. **(b)** The changes of ΔΨm represented as the ratio between red and green fluorescence of JC-1. **(c)** ATP levels were measured by luminescence. Data were normalized to the control as 100%. **(d)** Typical trace showing the inhibitory effect of compound **1** (2.5 mM) on NaT-induced ROS production (H_2_DCFDA, F/F_0_). **(e)** Quantification of ROS production induced by NaT with or without compound **1**.Values were represented as mean ± S.E.M. and n ≥ 3 per condition, ^#^ and * p < 0.05 vs control and NaT, respectively.

Since compound **1** at 2.5 mM showed a maximal inhibitory effect on necrotic cell death pathway activation induced by NaT. Mitochondrial dysfunction is one of the key regulator of cell death pathway activation, considered as ‘the point of no return’^21^. We next examined the effects of compound **1** at 2.5 mM on mitochondrial membrane potential. At high membrane potential, JC-1 forms red-fluorescent J-aggregates, whereas green-fluorescent monomer exists at low potential. Therefore, the ratio between red and green fluorescence is used to measure changes in ΔΨm. NaT resulted in a dramatic decrease of ΔΨm shown as decrease of red/green fluorescence ratio; application of compound **1** prevented the loss of ΔΨm, by 56.36% as compared to Na-T (Fig. 3a,b). Since ATP production through electron transport chain is mediated by ΔΨm and the levels of ATP is directly correlated with the severity of pancreatic injury^22^. We then tested the effects of compound **1** on ATP production. We showed that NaT caused a marked reduction of ATP production at 20 min and 40 min; compound **1** significantly prevented the decrease of ATP levels by 69.01% at 20 min and 83.21% at 40 min compared with NaT (Fig. 3c). Mitochondria are a major source of ROS through electron transport chain on the inner mitochondrial membrane^23^. We then evaluated the effect of compound **1** on cytosolic ROS level [ROS]_i_ using 2, 7-dichlorodi-hydrofluorescein diacetate (H_2_-DCFDA). 5 mM NaT caused a sustained increase in [ROS]_i_; pretreatment of compound **1** significantly decreased [ROS]_i_ by 32.22% (Fig. 3d,e). Taken together, our *in vitro* data suggested that compound **1** effectively protested against necrotic cell death pathway activation induced by NaT, a well-known pancreatitis-associated toxin that correlated with biliary AP. The protective effects are largely dependent on rescuing the cells from mitochondrial dysfunction and ATP depletion by inhibiting loss of ΔΨm and excessive ROS production.

### Protective effects of Compound 1 on three experimental models of AP

**Figure 4:**
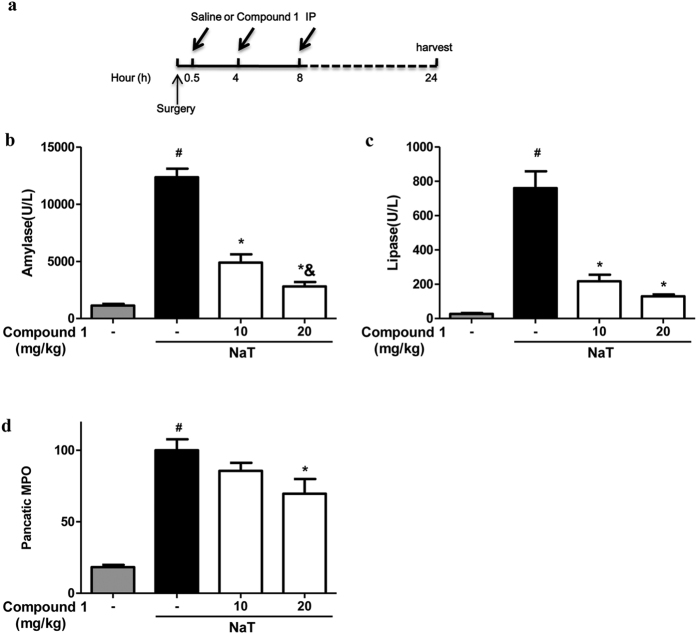
Compound 1 markedly reduced biochemical responses in NaT-AP. **(a)** Schema for administration of compound **1** after induction of pancreatitis by retrograde pancreatic ductal injection of 3.5% NaT. Retrograde pancreatic ductal injection of 3.5% NaT caused marked elevations of **(b)** serum amylase, **(c)** serum lipase, and **(d)** pancreatic myeloperoxidase (MPO). Intraperitoneal injection of compound **1** at the dose of 10 and 20 mg/kg significantly reduced all parameters with a more pronounced reduction at the high dose. Data were represented as mean ± S.E.M. and n = 6 per group. ^#^ and * p < 0.05 vs control and NaT, respectively; ^&^p < 0.05, low dose vs high dose.

**Figure 5:**
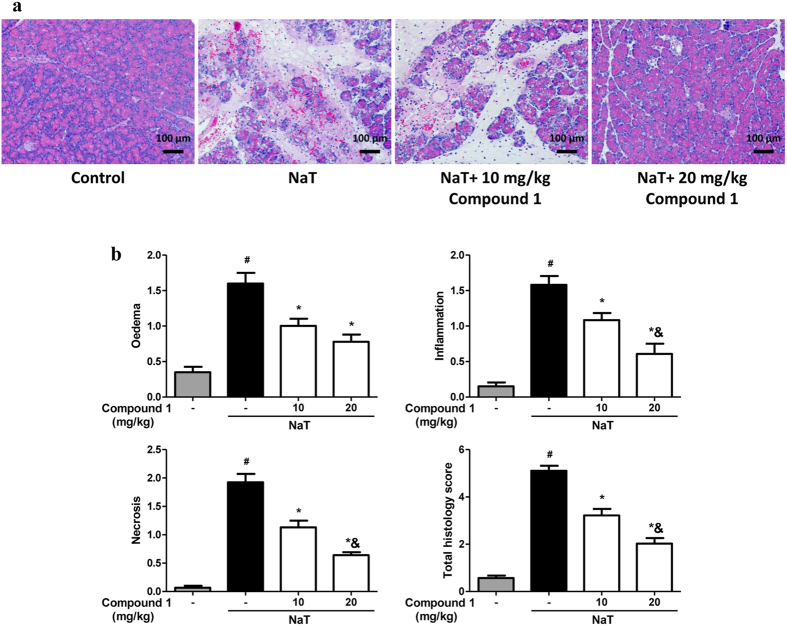
Compound 1 markedly reduces pancreatic histopathology in NaT-AP. **(a)** Representative haematoxylinand & eosin (H&E) section of pancreas from control, NaT, NaT treated with 10 mg/kg compound **1**, and NaT treated with 20 mg/kg compound **1**. **(b)** Blinded histopathological analysis for edema, inflammation, necrosis and total histological scores. Intraperitoneal injection of compound **1** at the dose of 10 and 20 mg/kg significantly reduced all score with a more marked reduction at the high dose. Data were represented as mean ± S.E.M. and n = 6 per group. ^#^ and * p < 0.05 vs control and NaT, respectively; ^&^p < 0.05, low dose vs high dose.

Since we demonstrated that compound **1** effectively protected from necrotic cell death pathway activation, and the severity of experimental AP correlates directly with the extent of necrosis^9^, we next investigated the effects of compound **1** in three murine models of AP. Firstly, we tested compound **1** in NaT-AP, which is representative of acute biliary pancreatitis from ampullary gallstone obstruction^24^ and was induced by retrograde pancreatic ductal injection of 3.5% NaT in rats^25^. Preliminary *in vivo* data indicated a half-life of these compound class is 4 h^26^, so it was intraperitoneally administered 0.5, 4 and 8 h after the induction (Fig. 4a). Compound **1** at 10 and 20 mg/kg reduced serum amylase, by 60.37% and 77.25% respectively and reduced serum lipase, by 71.41% and 83%, respectively (Fig. 4b,d). Pancreatic myeloperoxidase (MPO) activity was significantly reduced by 30.35% at high dose only (Fig. 4d). Consistently, compound **1** dose-dependently reduced pancreatic edema, inflammatory cell infiltration, acinar cell necrosis, total histological scores; inflammatory cell infiltration, acinar cell necrosis, and total histological scores were significantly more by high dose with acinar cell necrosis approaching the sham level (Fig. 5).

**Figure 6:**
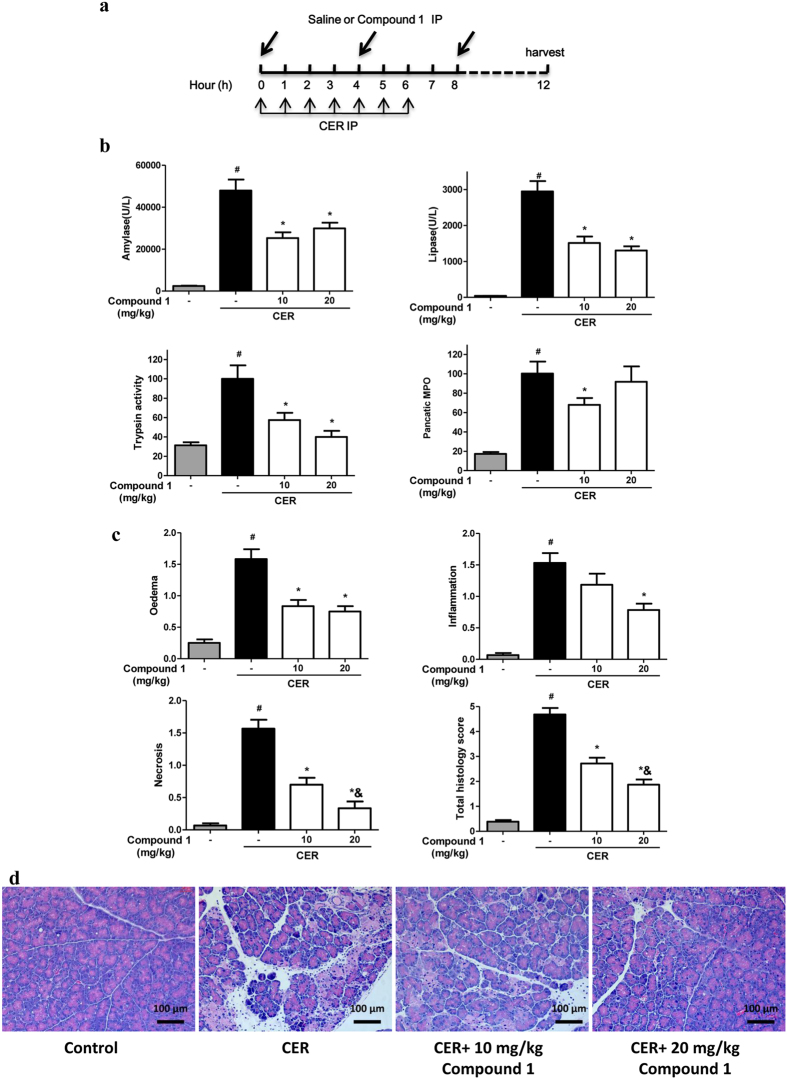
Compound 1 markedly reduced biochemical and histological pancreatic damage in CER-AP. **(a)** Schema for administration of compound **1** in CER-AP. Seven hourly intraperitoneal injection of CER caused a substantial increase of **(b)** amylase, lipase, pancreatic trypsin activity and pancreatic MPO activity as well as **(c)** blinded histopathological scores for edema, inflammation, necrosis and total histological scores. Intraperitoneal administration of 10 and 20 mg/kg compound **1** significantly reduced all biochemical and histological parameters. Data were represented as mean ± S.E.M. and n = 6 per group. ^#^ and * p < 0.05 vs control and CER, respectively; ^&^p < 0.05, low dose vs high dose.**(d)** Representative H&E section of pancreas from control, CER, CER treated with 10 mg/kg compound **1** and CER treated with 20 mg/kg Compound **1**.

Hyperstimulation AP induced by caerulein (CER) is the most widely used and highly reproducible model of AP^24^. Compound **1** was intraperitoneally administered along with 1^st^ and 5^th^ injections of CER, and 1 h after last injection of CER (Fig. 6a). At both doses compound **1** significantly reduced the increases of serum amylase, lipase, and pancreatic trypsin activity; pancreatic MPO activity was reduced significantly by low dose only (Fig. 6b). Consistently, compound **1** dose-dependently decreased pancreatic edema, inflammation, acinar cell necrosis, and total histological scores; acinar cell necrosis and total histological scores were significantly more by high dose, with acinar cell necrosis approaching to the control level (Fig. 6c,d).

**Figure 7:**
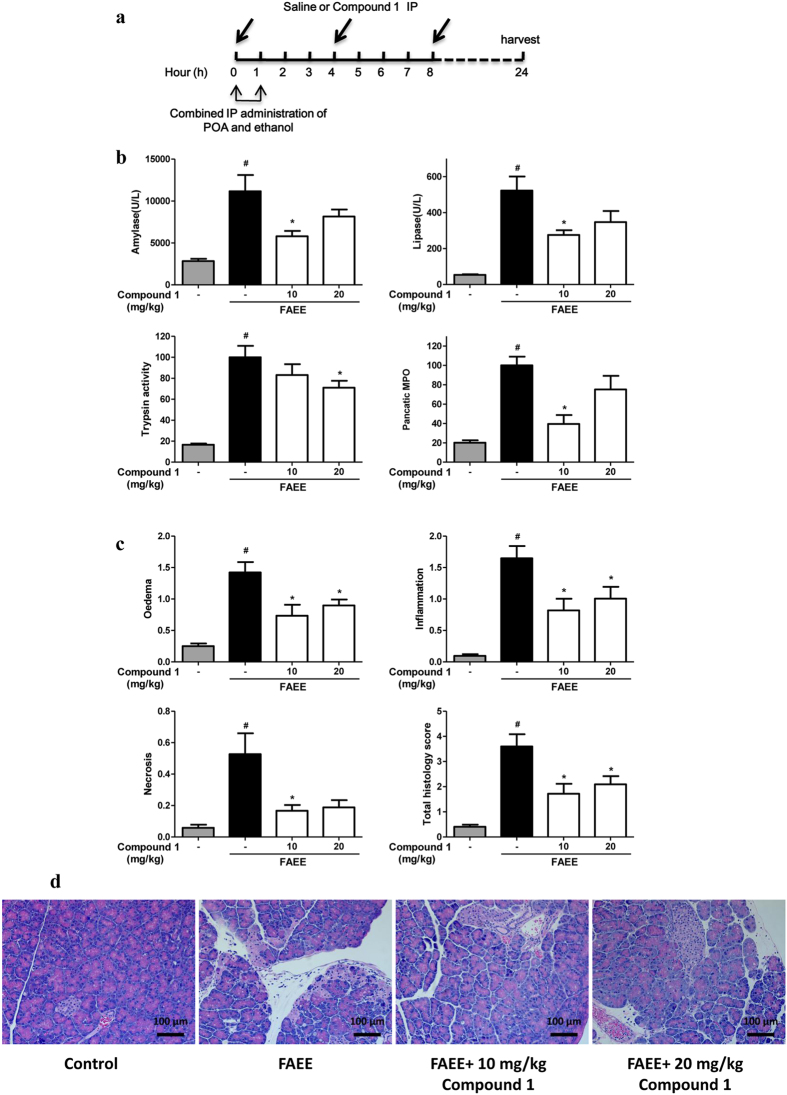
Compound 1 markedly reduced biochemical and histological pancreatic damage in FAEE-AP. **(a)** Schema for administration of compound **1** in FAEE-AP. Two hourly intraperitoneal injection of a mixture of palmitoleic acid and ethanol caused a substantial elevation of **(b)** amylase, lipase, pancreatic trypsin activity and pancreatic MPO activity as well as **(c)** blinded histopathological scores for edema, inflammation, necrosis and total histological scores. Intraperitoneal administration of 10 mg/kg compound **1** significantly reduced all biochemical and histological parameters with a trend towards decrease all at 20 mg/kg. Data were represented as mean ± S.E.M. and n = 6 per group. ^#^ and * p < 0.05 vs control and FAEE, respectively. **(d)** Representative H&E section of pancreas from control, FAEE, FAEE treated with 10 mg/kg compound **1** and FAEE treated with 20 mg/kg compound **1**.

Alcoholic fatty acid ethyl esters (FAEE-AP) mimics acute alcoholic pancreatitis through *in vivo* formation of toxic non-oxidative ethanol metabolites^27^, and was induced by intraperitoneal co-administration of palmitoleic acid (POA) and ethanol^28^. Compound **1** was intraperitoneally injected at the time of FAEE-AP induction, 4 and 8 h after the first dose (Fig. 7a). Compound **1** at 10 mg/kg significantly reduced serum amylase, lipase, and pancreatic MPO activity, but not pancreatic trypsin activity. However, compound **1** at 20 mg/kg significantly decreased pancreatic trypsin activity with a trend towards decrease in other biochemical parameters (Fig. 7b). At both doses compound **1** markedly reduced pancreatic edema, inflammatory infiltrate, acinar cell necrosis and total histological scores, with acinar cell necrosis approaching to the control level (Fig. 7c,d).

### Compound 1 protected against pancreatitis-associated acute lung injury

**Figure 8:**
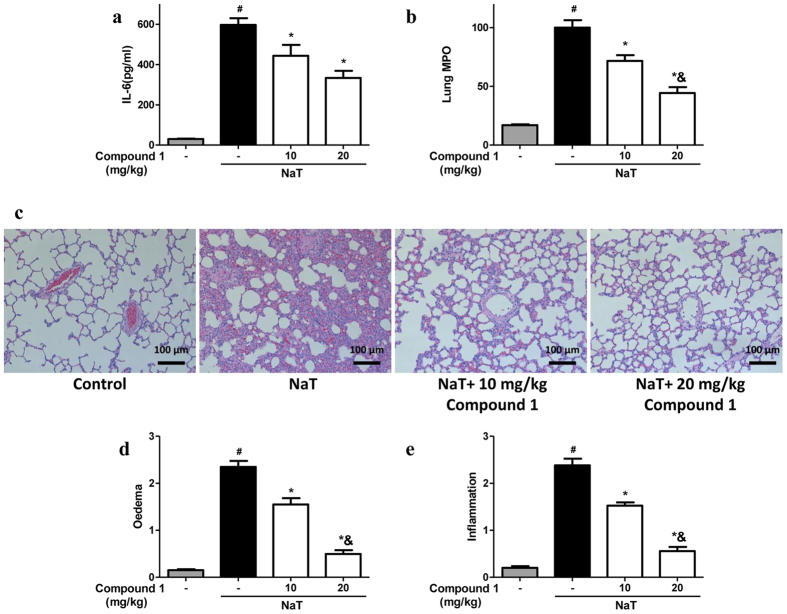
Compound 1 protected against pancreatitis-associated acute lung injury in NaT-AP. Retrograde pancreatic ductal injection of 3.5% NaT caused a dramatic lung injury assessed biochemically by **(a)** IL-6 and **(b)** lung MPO activity. Intraperitoneal administration of compound **1** at the dose of 10 and 20 mg/kg significantly reduced both markers with more pronounced reduction at the high dose. **(c)** Representative H&E sections of lungs from control, NaT, NaT treated with 10 mg/kg compound **1** and NaT treated with 20 mg/kg compound **1**. Lung histopathological analysis was blindly assessed by **(d)** edema and **(e)** inflammation. Data were represented as mean ± S.E.M. and n = 6 per group. ^#^ and * p < 0.05 vs control and NaT, respectively; ^&^p < 0.05, low dose vs high dose.

ALI is one of the commonest complications in severe AP and associated with significantly higher mortality^10^,^19^. Among three experimental models we used in this study, NaT-AP had more severe ALI overall as assessed by Interleukin-6 (IL-6), lung MPO and lung histology than hyperstimulation AP and FAEE-AP (Fig. 8, Supplementary Figs 1 and 2). Remarkably, compound **1** at 10 and 20 mg/kg dramatically reduced biochemical (IL-6 and lung MPO activity) and histological (edema and inflammatory scores) responses of ALI in NaT-AP (Fig. 8); more significant reduction by high dose in MPO activity, edema and inflammatory infiltrate; 55.69%, 78.94%, and 76.63% down to the control levels, respectively (Fig. 8b–e). In hyperstimulation AP induced by CER, compound **1** had no protective effects on IL-6 and lung MPO activity (Supplementary Fig. 1a,b). While compound **1** at high dose significantly reduced inflammatory infiltrate, by 48.08%, with a trend towards a decrease in edema at both doses and inflammatory infiltrate at low dose (Supplementary Fig. 1c–e). Similarly, compound **1** had no protective effects on IL-6 and lung MPO activity in FAEE-AP, with a trend towards a decrease at high dose (Supplementary Fig. 2a,b). Whereas compound **1** at low dose significantly reduced edema and inflammatory infiltrate, by 39.17% and 53.81%, respectively with a trend towards a reduction at high dose (Supplementary Fig. 2c–e).

## Discussion

In this study, we found five saponin compounds isolated from *Dioscorea zingiberensis* C. H. Wright protected against necrotic cell death pathway activation induced by NaT, a well-known pancreatitis-associated toxin. Among them, compound **1** exhibited the most consistent and pronounced protective effects on NaT-induced necrosis in pancreatic acinar cells in a concentration-dependent manner with the maximal inhibitory effect at 2.5 mM. Furthermore, compound **1** at 2.5 mM prevented the loss of ΔΨm, ATP depletion, and excessive ROS production^21^. Previous studies have shown that induction of mitochondrial permeability transition pore (MPTP), featured by sudden increased permeability in inner mitochondrial membrane, loss of ΔΨm and impaired ATP production, is the central mechanism of mitochondria-mediated necrosis and the key determinant of injury in various models of AP^29^. The severity of AP is directly correlated with the extent of necrosis and conversely with the degree of apoptosis in several experimental models of AP^9^,^22^. Saponins from *Panax quinquefolium* have been shown to prevent MPTP opening and cell injury in a rat myocardial ischemia/reperfusion model^30^. Avicins, a class of triterpenoid saponins, have been shown to promote apoptosis by inducing cytochrome c release, increasing caspase-3 activation and decreasing ROS generation in Jurkat human T cell line^31^. Consistently, our *in vitro* data highly suggested the protective effects of diosgenyl saponins, specifically compound **1** on preventing necrotic cell death pathway activation are mainly through mitochondrial protection.

Next, we showed the protective effects of compound **1** at 10 and 20 mg/kg on disease severity in three murine models of AP. Administration of compound **1** at the time of or after AP induction significantly reduced all biochemical and pancreatic histopathological parameters in all three models. Consistent with *in vitro* findings, compound **1** more dramatically reduced pancreatic necrosis assessed by histopathological scores than other histological components (edema and inflammation), suggesting the primary protective effects exerts within the pancreas. A recent study showed that pancreatic stellate cells are more sensitive to bile acid-induced pancreatic^32^, indicating the protective effects of compound **1** observed *in vivo* could be through pancreatic stellate cells. Previous studies showed that saponins isolated from natural products have anti-inflammatory effects in a wide range of inflammatory diseases. Steroids saponins isolated from *Smilax chinaL* exhibited anti-inflammatory effects on murine peritoneal macrophages via inhibition of cyclooxygenase-2 (COX-2) activities^13^. Akebia Saponin D protected against neuro inflammation via inhibiting the expression of COX-2, tumor necrosis factor -α and IL-1β as well as Akt/nuclear factor kappa B (NF-κB) activation^17^. Moreover, diosgenin inhibited macrophage-mediated inflammation through down-regulating of CK2, c-Jun NH_2_-terminal kinase (JNK), NF-κB and AP-1. Our *in vivo* data for the first time demonstrated that diosgenyl saponins, specifically compound **1** exhibited the *in vivo* protective effects primarily through inhibition of parenchymal cell injury in the targeted organ at the initiation of the inflammation.

Remarkably, compound **1** also significantly reduced the extent of pancreatitis-associated ALI in NaT-induced pancreatitis with a trend towards a reduction in other two models. During AP, the initial injury starts within pancreatic acinar cells, which makes up the bulk of the pancreas. Damaged parenchymal cells can produce, release, and respond to cytokines, leading to amplification of the inflammation locally and systemically. Distant organ damage is believed to be mediated by excessive immune response^33^,^34^. One of the critical signaling molecules, NF-κB, has been shown to play a central role in the development and amplification of inflammation during AP^35^. Shen-fu injection (ginseng saponin as its main component) ameliorated caerulein-induced AP by modulating oxidative stress and NF-κB activity^36^. Korean red ginseng, containing major ginseng saponins, protected against caerulein- or pancreatic duct ligation-induced AP via suppressing hydrogen sulfide production and inactivating NF-kB and JNK pathway^37^,^38^. Diosgenyl saponins may confer the protective effects on distant organ damage through regulating the key inflammatory pathways, such as NF-kB pathway.

In summary, this study for the first time showed that diosgenyl saponins extracted from *Dioscorea zingiberensis* C. H. Wright, particularly compound **1** protected against pancreatic acinar cell injury and experimental AP primarily through mitochondrial protection within the pancreas. Compound **1** offers a novel potential therapy for treating AP and serve as the starting point for further development. More broadly, this study suggests natural saponins may serve as fruitful sources for exploring/identifying potential therapies for the inflammatory diseases.

## Materials and Methods

### Animals

Ethical approvals for all experiments and methods had been obtained from the Ethics Committee of West China Hospital of Sichuan University (2014006B), and were performed in accordance with ARRIVE guideline^39^. Male Balb/C mice (25–30 g) and male Wistar rats (250–300 g) were used. Mice and rats were maintained in a climate-controlled room at 22 °C and exposed to a 12 h light/12 h dark cycle, fed with standard food and water, allowed to acclimatize for a minimum of one week. All efforts were made to minimize animal suffering and to reduce the number of animals used.

### Isolation and structural characterization of extracts

10 kg of dried and coarsely powdered rhizomes of *D. zingiberenesis* C. H. Wright was extracted three times with ethanol at room temperature for 24 h. The extract was filtered through Buchner funnel and concentrated using vacuum rotary evaporator at 40 °C. At the end 650 g of crude ethanol extract was obtained and partitioned between equal amounts of water and dichloromethane layers. The water layers was concentrated to obtain the extract and 230 g of this extract was chromate graphed on a macro porous resin column and successively eluted with stepwise gradient of 20% ethanol and 70% ethanol. 70% ethanol fraction was collected and concentrated. A light yellow color precipitate was obtained in this fraction and washed with methanol. Then the precipitate was dissolved in DMSO and subjected to preparative High Performance Liquid Chromatography (HPLC) (YMC-pack ODS-A C18, 20 mm × 10 mm, 5 μm; eluent, 55% acetonitrile; dection, UV at 210 nm; flow rate, 6 mL/min) to yield compounds **2**–**5**. The filtrate of 70% ethanol Fraction was concentrated and chromate graphed on a ODS column (500 mm × 50 mm, 50 μm) with a gradient elution of MeOH-H_2_O (5:95–100:0) to yield 5 fractions (A-E). Fraction A was subjected to MPLC (C18 column, 500 × 10 mm, 50 μm; detection, UV at 210 nm; 10 mL/min) with a gradient elution of methanol-H_2_O (20:80–50:50) to yield 5 fractions (A1-A5). Fraction A3 was subjected to HPLC (YMC-pack ODS-A C18, 250 × 10 mm, 50 μm; eluent, MeCN-H_2_O (25:75); detection, UV at 210 nm; flow rate, 6 mL/min) to yield compound **1**. The purities of **1**–**5** were determined by HPLC (Shimadzu LC-20A) equipped with a DAD detector. HPLC analysis was carried out on a reversed-phase C18 column (20 mm × 4.6 mm, 5 μm) maintained at room temperature with a flow rate of 0.8 ml/min. The HPLC analysis showed that the purities of **1**–**5** were >96%. The structures of the compounds were identified by ^1^H and ^13^C NMR spectra.

### Pancreatic acinar cell preparation

Isolation of pancreatic acinar cells from Balb/c mice was performed using a collagenase digestion procedure as described^28^,^40^,^41^. In brief, animals were sacrificed by cervical dislocation, pancreas was dissected, washed twice in phosphate-buffered solution (PBS), then injected with collagenase **IV** (200 U/mL) and incubated in water bath at 37 °C for 20 min. After the incubation, cells were isolated by mechanical dissociation, centrifuged at 700 rpm for 2 min to obtain cell pellet, and were suspended in the extracellular solution containing (in mM): 140 NaCl, 4.7 KCl, 1.13 MgCl_2_, 1 CaCl_2_, 10 D-glucose, and 10 HEPES (adjusted to pH 7.35 using NaOH). Cells were treated at room temperature, and used within 4 h after isolation.

### Necrotic cell death pathway activation measurement

Cells were treated with 5 mM NaT with or without various concentrations of compound **1–5** for 40 min, gently shaking at 50 rpm at room temperature for assessing necrosis^41^. After washing, cells were loaded with Hoechst 33342 (50 μg/ml) for staining the nuclei and counting total number of cells; propidium iodide (PI, 1 μM) for assessing plasma membrane rupture. Images were recorded by fluorescence microscopy ZEISS AX10 imager A2/AX10 cam HRC (Jena GmbH, Germany). The total number of cells showing PI uptake was counted from each condition, more than 1,000 total cells were counted, to provide a percentage (necrosis%). At the screening stage, the experiments were performed with pancreatic acinar cells isolated from 3 mice per condition, followed by another set of experiment mainly focusing on compound **1**, with pancreatic acinar cells isolated from 6 mice per condition.

### Mitochondrial membrane potential measurement

ΔΨm was measured by using JC-1-based mitochondrial membrane potential detection kit (Molecular Probes, Eugene, USA) according to manufacturer’s instructions. At high membrane potential, JC-1 forms red-fluorescent J-aggregates, whereas green-fluorescent monomer exists at low potential. The ratio between red and green fluorescence is used to measure changes in ΔΨm. Fresh cells were treated with 5 mM NaT ± 2.5 mM compound **1** for 30 min, washed twice in extracellular solution, then loaded with JC-1 at 37 °C avoiding from light for 10 min, washed twice in PBS, and the red (excitation 550 nm, emission 600 nm) and green (excitation 485 nm, emission 535 nm) fluorescence were measured by fluorescent microscopy (Imager Z2, Zeiss). Mitochondrial depolarization manifests by a decrease in the ratio of the red and green fluorescence. Image processing and analysis were performed by ImageJ software (ImageJ, NIH). The experiments were performed with pancreatic acinar cells from 3 different mice per condition.

### ATP measurement

ATP levels were measured as described^42^ using an ATP determination kit (Beyotime Biotechnology, Shanghai, China) according to manufacturer’s instructions. Briefly, cells were treated with 5 mM NaT ± 2.5 mM compound **1** for 20 and 40 min, washed twice in extracellular solution, centrifuged at 700 rpm for 2 min, re-suspended in lysis buffer, boiled for 2 min, centrifuged at 12000 g for 5 min. Luminescence in the supernatant from each sample was measured in a Synergy Mx multifunctional Microplate Reader (Gene Company Ltd, Hongkong, China). Data were normalized to protein concentration for each sample and, then normalized to the control as 100%, with pancreatic acinar cells isolated from 5 different mice per condition.

### ROS measurement

Cells were incubated with H_2_-DCFDA, a ROS indicator for 20 min, avoiding from light at 37 °C, then distributed into 96-well glass black plates after washing. Fluorescence was recorded by Synergy Mx multifunctional Microplate Reader for 30 min in total; baseline was recorded for 5 min, 2.5 mM compound **1** added and recorded for 5 min; then 5 mM NaT added and recorded for 20 min. The data were expressed as F/F_0_ and quantified as the ΔF/F_0_ at 2250 s with or without compound **1**. The experiments were performed with pancreatic acinar cells isolated from 3 different mice per condition.

### Models of AP

Mice or rats were fasting for 18 h before induction of AP. Biliary AP (NaT-AP) was induced in Wistar rats, by retrograde pancreatic ductal injection with 3.5% NaT (100 μL/min by infusion pump)^25^; killed at 24 h after the induction. Anesthesia was achieved with 30 mg/kg pentobarbital sodium. Compound **1** (10 mg/kg or 20 mg/kg) was administered 0.5, 4 and 8 h after the surgery. Rats received laparotomy only served as the controls. Hyperstimulation AP (CER-AP) was induced in Balb/c mice, by seven hourly intraperitoneal injections of CER (50 μg/kg); humane killing was made 12 h after the first injection of CER^24^. Compound **1** (10 mg/kg or 20 mg/kg) was administered at 0, 4 h and 8 h. Alcoholic AP (FAEE-AP) was induced by a modified method from Huang *et al*.^2^ Specifically, a mixture of 200 mg/kg POA and 1.75 g/kg ethanol was intraperitoneally injected twice at 1 h interval, 200 μL saline was injected before ethanol/POA injection to avoid potential damage induced by ethanol; humanely killed 24 h after the induction. Compound **1** (10 mg/kg or 20 mg/kg) was administered at 0, 4 h and 8 h after the first injection of POA and ethanol. Mice received equal volume of normal saline with the corresponding injections served as the controls.

### Serum amylase, lipase and IL-6

Blood was collected by cardiac puncture and centrifuged at 3500 rpm for 15 min. Serum amylase and lipase was measured using a full automatic biochemical analyzer (Roche, Mannheim, Germany). IL-6 levels were evaluated by IL-6 ELISA kit (Abcam, Cambridge, United Kingdom) according to the manufacturer’s protocol.

### Histology

Specimens of murine pancreas and lung were fixed in 10% formaldehyde overnight. Tissues were embedded in paraffin, sectioned in 5 mm and stained with haematoxylin and eosin. Pancreatic histopathological scores were evaluated blindly by two pathologists for edema, inflammatory cell infiltration and necrosis, from 0 to 3 as previously described^43^. Lung histopathological scores were evaluated for edema and inflammatory cell infiltration, from 0 to 3 as previously described^44^.

### MPO activity

Pancreatic and lung MPO activity was measured by a modified method from Dawra *et al*.^45^ Briefly, pancreatic and lung tissue was homogenized on ice in 100 mM phosphate buffer (pH 7.4) containing protease inhibitors, then resuspended in 100 mM phosphate buffer (pH 5.4) containing protease inhibitors. The suspension was freeze-thawed in three cycles, sonicated for 30 seconds. 20 μL of the supernatant was added into the assay solution, consisting of 200 μL of phosphate buffer (100 mM, pH 5.4) with 0.5% hexadecyltrimenthyl ammonium bromide (HETAB), 20 μl 3,3,5,5,-tetramethylbenzidine(TMB, 20 mM in DMSO), incubated at room temperature for 3 min, followed by the addition of 50 μL H_2_O_2_ (0.01%). The absorbance difference at 650 nm between 0 min and 3 min was measured at 37 °C with a Synergy Mx multifunctional microplate reader, normalized to protein concentration for each sample, then normalized to AP group as 100 for each model.

### Pancreatic trypsin activity

Trypsin activity was measured in homogenized pancreatic tissue as described previously^46^. Briefly, pancreata were homogenized on ice in a buffer containing 5 mM MOPS, 250 mM sucrose and 1 mM magnesium sulphate (pH 6.5). The homogenates were centrifuged at 1500 g for 5 min, and 10 μL of each supernatant was incubated at 37 °C for 300 s in 280 μL assay buffer containing 50 mM Tris-HCl (pH 8.0), 150 mM NaCl, 1 mM CaCl_2_, and 0.1 mg/ml bovine serum albumin and peptide substrate Boc-Gln-Ala-Arg-MCA. Trypsin activity was measured by fluorimetric assay. Fluorescence was recorded (excitation 380 nm and emission 440 nm) and data were normalized to protein concentration for each sample; then normalized to AP group as 100% for each model.

### Materials

Collangenase **IV** was purchased from Worthington Biochemical Corporation (Lakewood, NJ, USA). Hoechest33342, PI and H_2_-DCFDA were from Molecular Probes (Eugene, Oregon, USA). Taurocholic acid sodium salt hydrate (T4009), caerulein (C9026), palmitoleic acid (P9417), hexadecyltrimenthyl ammonium bromide and 3,3,5,5,-tetramethylbenzidine were purchased from Sigma-Aldrich (St. Louis, MO, USA). Boc-Gln-Ala-Arg-MCA (600318) was purchased from Peptide (Ibaraki, Osaka, Japan). Protease inhibitor cocktail tablets (118361700001) were from Roche Diagnostics (Mannheim, Germany). All other chemicals were purchased from Sigma-Aldrich unless stated (St. Louis, MO, USA).

### Statistical Analysis

Data were presented as mean ± SEM, statistical analyses were performed by using one-way analysis of variance (ANOVA) followed by 2-tailed Students’t- test when appropriate. P < 0.05 was considered significant.

## Additional Information

**How to cite this article**: Zhang, R. *et al*. One compound of saponins from *Disocorea zingiberensis* protected against experimental acute pancreatitis by preventing mitochondria-mediated necrosis. *Sci. Rep*. **6**, 35965; doi: 10.1038/srep35965 (2016).

## Supplementary Material

Supplementary Information
