# Supplementary material for: One compound of saponins from Disocorea zingiberensis protected against experimental acute pancreatitis by preventing mitochondria-mediated necrosis
Source: Sci Rep. 2016 Oct 25;6:35965. doi: 10.1038/srep35965 (PMC5078795; doi:10.1038/srep35965)
Supplement: Supplementary Information [file srep35965-s1.pdf]

One compound of saponins from *Disocorea zingiberensis* protected against experimental acute pancreatitis by preventing mitochondria-mediated necrosis

Rui Zhang[1,+], Li Wen[2,3+], Yan Shen[1], Na Shi[3], Zhihua Xin[1], Qing Xia[3*], Hai Niu[1,4*], Wen Huang[1*]

Supplementary Figure 1.

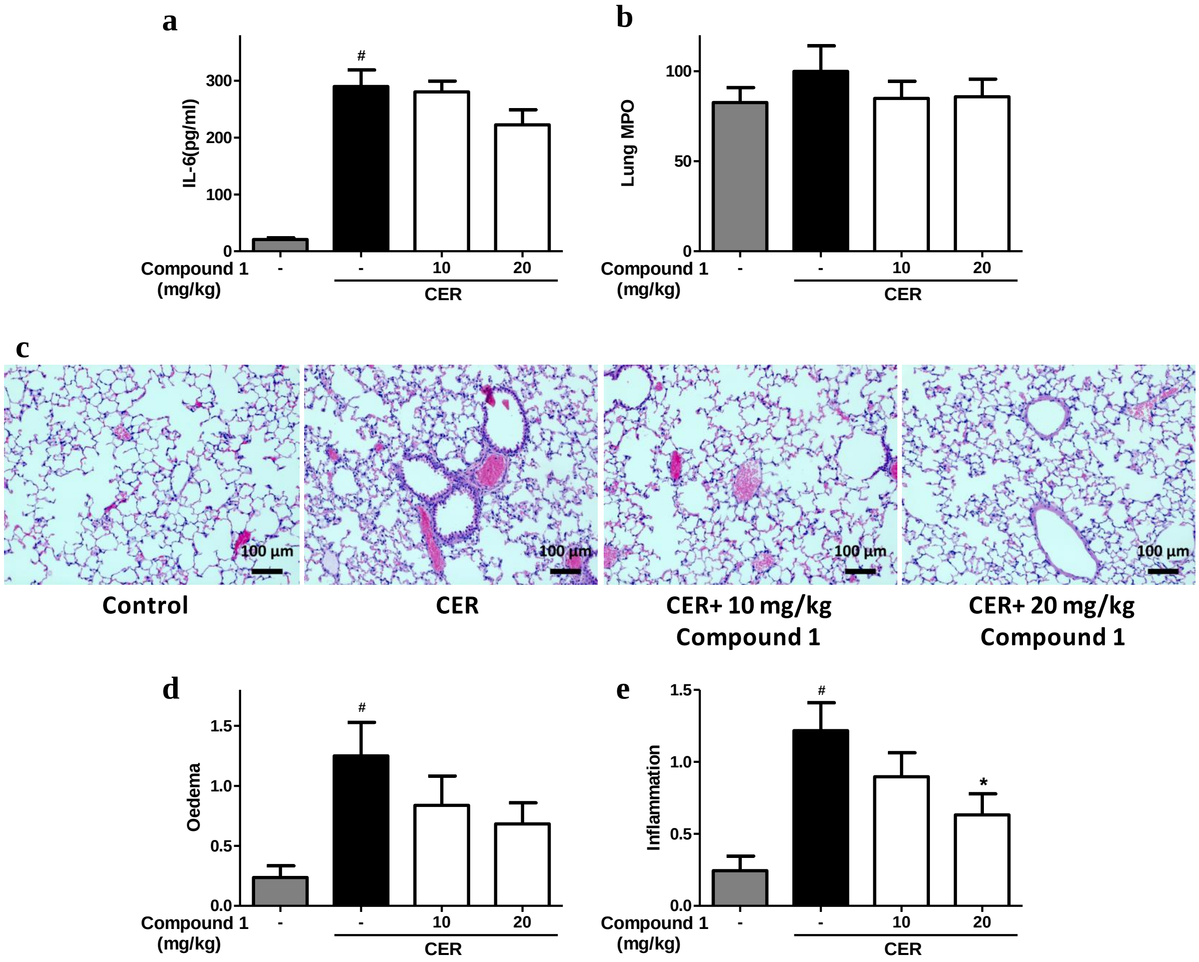

Supplementary Figure 1. Effects of compound 1 on pancreatitis-associated acute lung injury in CER-AP. Seven hourly intraperitoneal injection of CER caused an elevation of **(a)** IL-6 and **(b)** lung MPO activity. **(c)** Representative H&E lung sections from control, CER, CER treated with 10 mg/kg Compound **1** and CER treated with 20 mg/kg compound **1**. Lung histopathological analysis was blindly assessed by **(d)** edema and **(e)** inflammatory infiltrate. Data were represented as mean ± S.E.M. and n = 6 per group. $^{\#}$and * $p < 0.05$ vs control and CER, respectively.

Supplementary Figure 2.

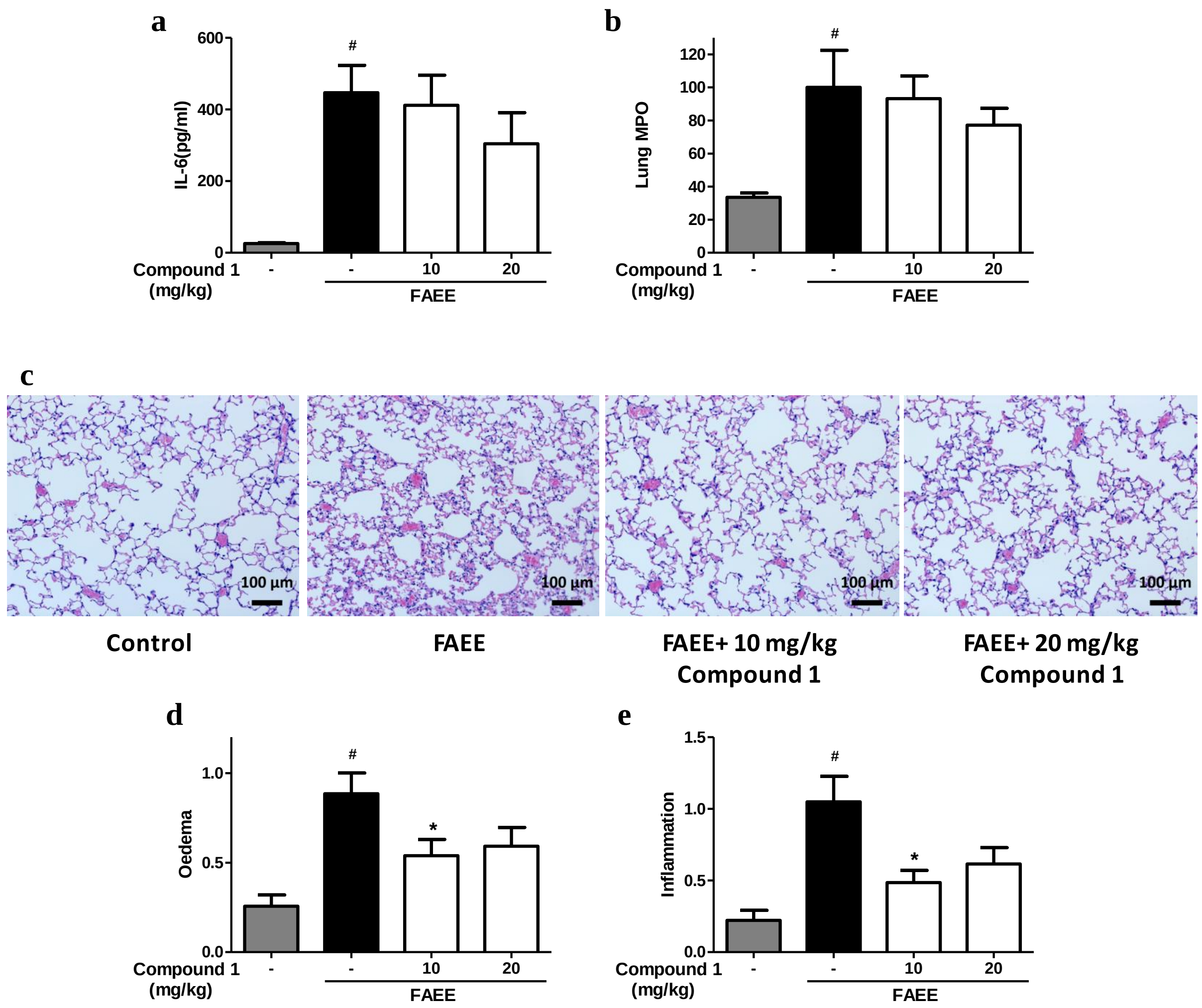

Supplementary Figure2. Effects of compound 1 on pancreatitis-associated acute lung injury in FAEE-AP. Two hourly intraperitoneal injections of POA and ethanol caused an elevation of **(a)** IL-6 and **(b)** lung MPO activity. **(c)** Representative H&E lung sections from control, FAEE, FAEE treated with 10 mg/kg compound **1** and FAEE treated with 20 mg/kg compound **1**. Lung histopathological analysis was blindly assessed by **(d)** edema and **(e)** inflammatory infiltrate. Data were represented as mean ± S.E.M. and n = 6 per group. $^{\#}$and * $p < 0.05$ vs control and FAEE, respectively.
